# The New York System of Treatment

**Published:** 1913-04-19

**Authors:** 


### THE SCHOOL CLINIC.

### II.—The New York System of Treatment.

The disadvantage under which our English system of school medical inspection suffers is that we make no provision for the treatment of defects found. The various local bodies have struggled with this problem of treatment and have attempted to deal with it in various ways. The London County Council, whose school work is more interesting than that of any other local body, simply because it deals with a far larger number of children, has compromised in accepting a partly hospital and partly privately staffed clinic system. This compromise does not work at all well. Anyone who has had experience of the way in which defective school children are treated in the out-patient departments of London hospitals and at the new clinics privately managed, cannot fail to have been struck with the relatively small percentage of cases in which the treatment has been thorough and efficacious. Another great drawback has been the difficulty of inducing parents to get their children treated for minor defects, such as carious teeth. At present it is doubtful (we speak subject to correction, of course) if more than 10 per cent. of cases found defective at the routine inspections are treated before the next inspection falls due.

### A Lesson in Following up Cases.

The New York report, chronicles what is apparently a much better state of things existing on the other side of the Atlantic. According to the *Monthly Bulletin* of the Department of Health of the 166,368 children reported defective in the previous year no fewer than 86 per cent. have had treatment. We are not told if the treatment has been successful, but the fact that 65,150 children have been taken by their parents to get treatment, in consequence of the advice of the school doctors and nurses, is a gratifying proof that in America the parents are not so apathetic as they appear to be here. We must bear in mind, however, that the American school nurse is much more of a mentor and family counsellor than her English colleague. She can herself take the child to be treated, if the parents will allow it, and during her daily school visits she can continue to urge the desirability of getting treatment. What is more, in certain cases, "if no treatment is obtained and the child's family is too poor to employ a physician, the nurse treats such cases in the school, following the procedure outlined by the Department of Health." This, however, affects only contagious defects. "Non-contagious defects are never treated at the schools. Nurses are under positive orders from the Department of Health not to give any treatment in non-contagious diseases, except first aid in emergencies. The nurse tells the children what is the nature of their defects as diagnosed by the physician, and advises them to see a physician. If this fails she explains the situation to the parents." Her most important work, therefore, lies in "following up" cases. At present there are far too few nurses, so that each nurse has too large a number of children under her charge. That is also the case with us, and the authorities should be urged to appoint more nurses, and to allow them more time for follow-up cases than they have at present. With us this duty of "following up" is delegated to voluntary workers on the Care Committees. The scheme does not work well, and the American plan seems better, if the nurse is allowed more time to do her home visiting. It is interesting to note that in America, too, both nurses and doctors complain of the amount of clerical work thrown on their shoulders. We have always thought that this clerical work, which is largely the collation of statistical data, should be done not by the nurses or doctors but by a properly trained clerical staff employed at the central divisional offices. Such a division of labour will ensure uniformity in collating the statistics, and rid the nurses and inspectors of a labour that threatens to swamp their more pressing duties and that robs them of much of the pleasure and interest that they ought to take in their school work.

We note, too, that under the present system the New York doctors no longer consult with parents at the examinations, as their English colleagues invariably do. This seems a mistake. The presence of the parent at the examination, if it can be secured, is always valuable to the doctor. He is in a position to ask questions concerning the child's previous history, and to gain information which would otherwise not be available first hand. Yet the American plan, perhaps owing to the fact that the nurses have so large an influence, has worked well. "The co-operation of parents in the school medical work," says the report, "is very satisfactory on the whole, although not so effective as it was during the first year." This is due, we venture to think, to the discontinuance of the consultations with the doctor at the school, but the report goes on to say that "at that time the parents believed treatment to be compulsory; now they are aware that they need not have their children treated unless they wish to. This knowledge detracts from good results. In many instances it was found that, while parents were willing to have their children treated, they were unable to give the time necessary for attendance at a dispensary for that purpose. At the written request of the parents a nurse accompanies the child to a dispensary." That, as Dr. Stevens pointed out some time ago in his Australian report, is a distinct advantage, and it will be well worth while to make similar provision for London children.

Dealing with the treatment itself the report refers to the work done at hospitals and dispensaries. And it is interesting, in this connection, to note that the strictures passed by Dr. Kerr on some London institutions seem to apply equally to New York hospitals so far as the treatment of defective school children is concerned. "Private physicians and dispensaries lack co-operation and take little interest in the work, which is detrimental to efficiency, as the Department of Health considers a case closed when it receives notice from a physician that the child is under his treatment," says the report.

A Grave Abuse in New York.

" The Department does not question the fact that a child is under medical care if a statement to this effect, signed by a physician, is presented. All the children presenting certificates of treatment are re-examined by the medical inspector, who notes on the child's record, and forwards to the Department a statement as to whether in his opinion such treatment is adequate. There is no law by which the Department can enforce any further action. The Department has information *that some physicians take advantage of this and carry on a trade in certificates sold at ten cents apiece* (the italics are ours). A certain druggist is said to be using pads signed by a physician and selling them as certificates for school purposes. He is said to advertise these certificates at moving-picture shows. Another physician in the Bronx had certificates printed and sold them at 25 cents (1s.) apiece! " We must admit that we have not progressed to that extent in England. " Out-patients' departments of hospitals, dispensaries, and clinics," the report proceeds, " co-operate very little in reporting the results of treatment, although they have been asked many times to do so, and special blanks for reporting were distributed among them. The need for free dental clinics is very urgent, as there is great lack of free dental facilities for the poor."*

The report contains a table giving the percentage of cases treated in 1911. From this it appears that 37,986 children were (satisfactorily?) treated by private practitioners and 27,164 (? satisfactorily) by hospitals. About an equal number of refraction cases were treated by hospitals and private practitioners. More than 58 per cent. of cases were reported to be under the care of private practitioners: in view of the revelations made previously it may be doubted whether this percentage were getting adequate treatment. Some 6,431 cases refused treatment, and 5,352 children were discharged from school and apparently lost sight of. From these figures it is evident that the percentage of children treated (or reported treated, which is rather a different matter) is higher in New York than in London. We regret, however, that the report gives no indication of the nature of treatment and does not state in how many cases such treatment was adequate. Nor do the signatories recommend that anything should be done in the way of establishing treatment centres. Apparently there is no machinery for starting such centres, and there is no money to equip and run them. We do not think that New York will continue to tolerate this haphazard fashion of dealing with her school children. She has admirable opportunities for starting her own treatment centres, and the report fully shows that much has to be done yet before the facilities for free dental treatment are what they should be. There is ample scope in this direction for American philanthropy and business methods. The report further deals with the health conditions of the children and with school hygiene generally. It points out the need for medical inspection in the higher-grade schools, and gives some startling information regarding the position of school caretakers or janitors.

The Janitor System.

It appears that the New York schools are farmed out to janitors, who are under a supervisor, but form an independent body or union of their own. This system, it is needless to say, opens the door to all sorts of abuses, and should be abolished as soon as possible. The question of school ventilation is touched upon. Physical drill and exercises are favourably reported on, and it is stated that the number of shower-baths in the higher-grade schools average ten to each school. In the lower-grade schools heating and ventilation " are so poor as to be a matter of great concern." Finally, the committee makes a number of important and interesting recommendations, with some of which we have already dealt. The committee urges that the number of children per nurse and doctor should be decreased; no physician should have charge of more than 7,500 and no nurse of more than 2,500. With that we fully agree, but it is a counsel of perfection which is not likely to be followed in the near future, especially in a city where economy seems to be the guiding principle in school medical inspection. Another recommendation is that the salaries of the nurses should be graded, starting at 800 dollars and rising to 1,000 dollars per year. This, as the report says, will undoubtedly act as a stimulus to efficient work. Compared with London nurses the New York school nurses are well paid, and it is possible that the latter can save more than the former can, though we are not told if they receive a retiring pension, as is the case with London County Council nurses. Further, it is recommended that " the medical practitioners and the dispensaries should be impressed with the importance of this work (of school medical inspection) to the community, and be urged to co-operate. Provision for dental clinics should be made, this being done if possible through the existing dispensaries." The committee urges " the establishment of a special bureau of school hygiene which should undertake the control of all the work."

We have dealt at some length with this interesting report, because it appears to us that the questions it raises are questions which concern us greatly at present. From a study of the report those of us who are interested in medical work in the schools may learn much, both with regard to the difficulties in the way of obtaining success and the manner in which they may possibly be overcome. Conditions in America are not, of course, similar to conditions here, but there is a close similarity between the problems in child life all the world over, and the best results can only be obtained by collating and studying the data which have been gathered from all quarters.

* All other quotations are given exactly in the language used in the report, a summary of which is to be found in the *New York Medical Record* of August 31, 1912, but in this instance it appeared justifiable to take the fair meaning and omit the word "to" before "co-operate" which makes the sentence unreadable.

